# Insights into the mechanisms of infection transmission via inanimate surfaces

**DOI:** 10.3389/fmicb.2026.1895041

**Published:** 2026-07-30

**Authors:** Katia Iskandar, Loïc Marchin, Christine Roques

**Affiliations:** 1 Faculty of Public Health-Section 2, Lebanese University, Fanar, Lebanon; 2 Center for Collaborative Research Initiatives in Public Health, Higher Institute of Public Health, Saint Joseph University of Beirut, Beirut, Lebanon, Beirut, Lebanon; 3 INSPECT-LB (Institut National de Santé Publique, d’Épidémiologie Clinique et de Toxicologie-Liban), Beirut, Lebanon; 4 Pylote SAS, Drémil-Lafage, Toulouse, France; 5 Laboratoire de Génie Chimique, CNRS, INPT, UPS, Faculté de Pharmacie, Université de Toulouse, Toulouse, France; 6 ACM Pharma Fonderephar, Canal Biotech, Toulouse, France

**Keywords:** biofilms, colonization, environmental reservoirs, fomites, healthcare-associated infections, infectious risk, microbial contamination, surface contamination

## Abstract

Inanimate surfaces are critical reservoirs for pathogenic microorganisms, increasing the risk of infectious disease transmission across healthcare, household, and public settings. This comprehensive review synthesizes current evidence on microbial contamination mechanisms, examining the complex interplay between pathogen characteristics, surface properties, and environmental conditions that govern fomite-mediated transmission. Contamination sources are diverse, originating from human shedding, respiratory secretions, environmental reservoirs, including airborne particles and water drainage systems, as well as contaminated materials such as medications, medical devices, and personal items. Pathogen persistence on surfaces ranges from hours to months, influenced by microorganism-specific attributes such as biofilm formation capacity, spore production, and structural characteristics that distinguish bacterial, viral, and fungal species. Environmental parameters, including temperature, relative humidity, pH, and light exposure, influence patterns of survival. Moisture-rich environments, in particular, enable persistence of Gram-negative bacteria and biofilm development. Surface characteristics, notably porosity, roughness, and material composition, create distinct microenvironments affecting microbial adhesion and persistence. Non-porous surfaces such as stainless steel and plastic generally support extended bacterial viability, whereas porous materials exhibit complex, pathogen-dependent survival patterns. Evidence from experimental studies, epidemiological investigations, and mathematical modeling confirms that inanimate surfaces are significant transmission vectors, particularly in healthcare settings where they contribute to 20–40% of healthcare-associated infections. Critical knowledge gaps persist regarding viable but non-culturable organisms, real-world transmission dynamics, and optimal cleaning/disinfection strategies. Future research priorities include developing advanced detection methods, refining cleaning and disinfection protocols, validating antimicrobial surface technologies, and establishing predictive models of transmission.

## 1 Introduction

Inanimate surfaces are not mere passive carriers of microorganisms (Kramer et al., 2006; Wißmann et al., 2021; Katzenberger et al., 2021; Jabłońska-Trypuć et al., 2022; Porter et al., 2024; Kramer et al., 2024). They are dynamic microbial reservoirs (Stephens et al., 2019; Wißmann et al., 2021; Katzenberger et al., 2021; Kramer et al., 2024; Jabłońska-Trypuć et al., 2022; Porter et al., 2024), serving as intermediaries in the transmission of pathogenic microorganisms and facilitating the spread of infectious diseases across different settings (Boone and Gerba, 2007; Stephens et al., 2019). Advances in molecular sequencing uncovered these surfaces as complex ecosystems (Kramer et al., 2024; Stephens et al., 2019), harboring microorganisms that can persist, adapt, and thrive through morphological, biochemical, and genetic changes, particularly within biofilms or as bacterial spores, and metabolic reduction (Stephens et al., 2019; Katzenberger et al., 2021; Kramer et al., 2024). Pathogens, including Gram-positive bacteria (GPB), Gram-negative bacteria (GNB), viruses, and fungi, can survive on dry surfaces for weeks to months (Kramer et al., 2006; Jabłońska-Trypuć et al., 2022; Kramer et al., 2024; Porter et al., 2024). Their persistence is influenced by numerous factors, including predominantly environmental conditions, surface characteristics, the types of microorganisms, and hygiene procedures (Kramer et al., 2006; Wißmann et al., 2021; Jabłońska-Trypuć et al., 2022; Kramer et al., 2024; Porter et al., 2024). High-touch surfaces in healthcare and community settings play a critical role in disease transmission, particularly for multidrug-resistant organisms (MDROs) (Kanamori et al., 2017; Weber et al., 2013a,b). Surface contamination encompasses a range of inanimate surfaces, including high-touch environmental surfaces, medical equipment, shared objects or devices, and personnel items. They serve as vehicles for microbial transmission (i.e., fomites) where potential pathogens demonstrate varying persistence patterns and infectious dose requirements across different materials and conditions (Levin et al., 2009; Katzenberger et al., 2021; Porter et al., 2024). Research indicated that most studies have evaluated microbial contamination of inanimate surfaces in healthcare settings, predominantly because fomites are a significant contributor to healthcare-associated infections (HAIs) (Bailey et al., 2019; Wißmann et al., 2021), especially in intensive care units (ICUs) and neonatal intensive care units (NICUs) (Kanamori et al., 2017). In community settings, monitoring is less stringent, despite the importance of shared surfaces in public transport systems schools, and workplaces (Jaydarifard et al., 2024). Recent research integrating experimental findings, mechanistic models, and epidemiological approaches has shed light on microbial interactions with fomites (Stephens et al., 2019; Porter et al., 2024). However, significant gaps remain in understanding the dynamics of fomite-mediated transmission. Current data predominantly originate from controlled laboratory studies that may not accurately reflect real-world conditions, indicating the need for more representative environmental investigations. (Stephens et al., 2019; Jabłońska-Trypuć et al., 2022; Kramer et al., 2024; Porter et al., 2024).

Beyond understanding contamination dynamics, mitigation strategies are increasingly explored. Emerging interventions include antimicrobial surfaces (Feuillolay et al., 2018; Querido et al., 2019; Iskandar et al., 2023), green biocides (Tong et al., 2024), and improved cleaning protocols (Boyce, 2016). These strategies can be complementary to traditional infection prevention and control (IPC) measures aiming to reduce microbial persistence and transmission.

By synthesizing current knowledge and updates on microbial contamination mechanisms through surfaces and examining the interplay between microorganism diffusion, surface properties, environmental conditions, replication capacity, and minimal infectious doses, this comprehensive review provides a refreshed overview of the latest trends in pathogen transmission dynamics in healthcare and community settings. These findings provide relevant insights for developing targeted interventions to mitigate fomite-born transmission and reduce the risk of infections.

## 2 Sources of microbial contamination of inanimate surfaces

**Figure 1:**
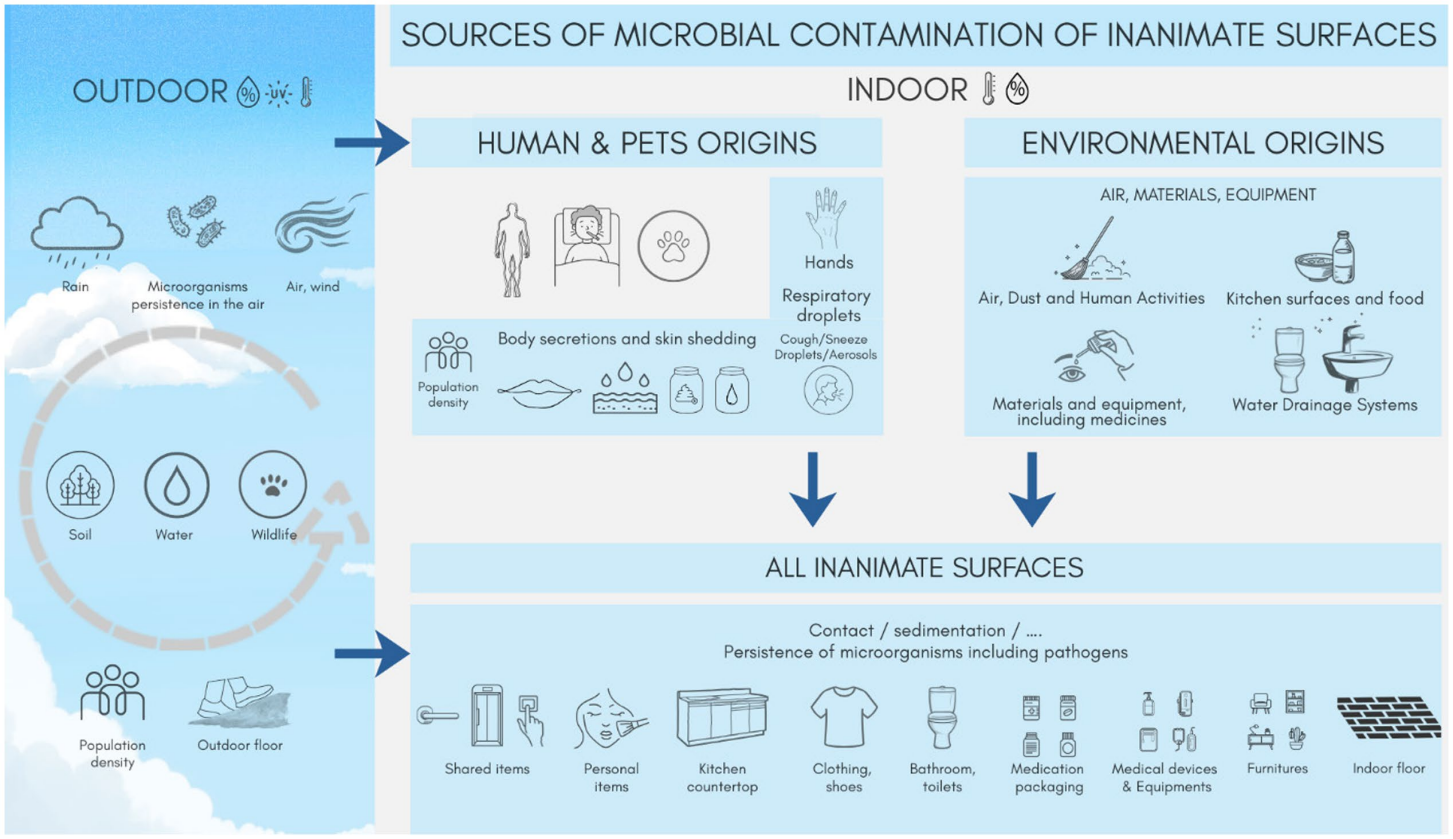
Sources of microbial contamination of inanimate surfaces. Original conceptual figure created by the authors using Canva. This schematic illustrates the pathways through which microbial contamination occurs. Outdoor sources such as air, wind, rain, soil, and water contribute to indoor contamination via airborne particles and human interactions. Indoor sources originate from human, pets and environmental origins. These vectors lead to contamination of inanimate surfaces through contact, sedimentation, and aerosolization, affecting shared surfaces, personal items including personal electronic devices and other items for personal use, kitchen surfaces, clothing, bathrooms, medication packaging, medical devices and equipment.

As illustrated in Figure 1, microbial contamination of inanimate surfaces arises from both outdoor and indoor sources. While the outdoor component provides the broader context, this section will focus on the indoor aspect, offering a detailed breakdown of its components and functionality. Indoor contamination is further categorized into human, animal and environmental origins, each contributing through distinct pathways to microbial contamination across various settings including household, healthcare and community (Table 1).

**Table 1:** Examples of surfaces prone to microbial contamination across different settings.

Category	Examples
Households’ surfaces prone to microbial contamination	
All furniture surfaces	All
Kitchen surfaces	Countertops, cutting boards, sinks, faucets, handles
Cooking equipment	Stove knobs, microwave buttons, blender bases, can openers
Food storage	Refrigerator shelves, pantry shelves, food containers, spice jars
Dining items	Plates, utensils, cups, straws, placemats
Cleaning tools	Sponges, dishcloths, mops, buckets, trash cans, kitchen towels
Bathroom surfaces	Toilet seats, faucets, showerheads, bathtub edges, towel racks, towels
Personal care items	Toothbrushes, razors, hairbrushes, soap dispensers, loofahs
Personal electronic devices	Tablets, mobile phones
Laundry items	Washing machine drums, dryer lint traps, laundry baskets, ironing boards
Living room surfaces	Remote controls, light switches, coffee tables, gaming controllers,
Bedroom surfaces	Bed linens, pillows, mattresses, nightstands, closet handles
Electronics	Smartphones, tablets, laptops, keyboards, TV remotes
Pet Items	Food bowls, water dishes, pet toys, litter boxes, pet bedding
Children’s items	Toys, high chairs, crib rails, pacifiers, sippy cups
Entryway surfaces	Doorknobs, shoe racks, keys, mail, umbrella stands
Outdoor items	Garden tools, patio furniture, grill surfaces, doormats
Medication containers	Eye drop bottles, nasal spray bottles, inhalers, pill organizers
Medical devices	Thermometers, blood pressure cuffs, glucose meters, breast pump parts
Aerosol-generating items	Nebulizers, CPAP machines, humidifiers, face masks (reusable)
First aid supplies	Bandages, tweezers, scissors, ointment tubes, hot/cold packs
Contact lenses and care	Contact lens cases, solution bottles, lens applicators
Personal hygiene	Menstrual cups, reusable pads, enema kits, ear syringes
Baby care items	Baby bottles, pacifiers, teething toys, diaper cream tubes
Drainage systems and water infrastructure	Sink siphons, drain traps, shower heads
Healthcare settings surfaces in prone to microbial contamination	
*Medical devices/shared equipment*	
Diagnostic equipment	Stethoscopes, otoscopes, ultrasound probes
Monitoring devices	BP cuffs, pulse oximeters, ECG leads
Treatment equipment	IV poles/pumps, feeding pumps, suction devices
Respiratory equipment	Ventilators, nebulizers, oxygen masks
Lab equipment	Centrifuges, analyzers, specimen containers
Imaging equipment	X-ray machines, portable units, ultrasound wands
Therapy equipment	Physical therapy tools, walkers, wheelchairs
*Patient care items*	
Bedding	Sheets, pillows, blankets, mattresses
Personal care	Bedpans, urinals, wash basins, commode chairs
Hygiene items	Toothbrushes, combs, razors, soap dispensers
Mobility aids	Canes, walkers, transfer belts
Patient clothing	Gowns, robes, slippers
Feeding items	Cups, straws, food trays, pitchers
Personal belongings	Storage bedside tables, closets
*Environmental surfaces*	
Bed components	Rails, controls, call buttons
Room surfaces	Doorknobs, light switches, window sills
Bathroom fixtures	Toilets, sinks, shower heads, grab bars, toilet flushing mechanisms
Furniture	Chairs, tables, visitor seating
Storage units	Cabinets, shelves, supply carts
Wall-mounted equipment	Hand sanitizer dispensers, glove boxes
Floor surfaces	Especially near patient beds, laundry and bathrooms
*Shared electronic devices*	
Communication devices	Phones, pagers, intercoms
Computer equipment	Keyboards, mice, tablets
Documentation stations	Charging stations, docking areas
Entertainment systems	TV remotes, patient tablets
Medical record devices	Barcode scanners, mobile workstations
Security devices badge readers, keypads	
Monitoring screens and controls	
*Staff items*	
Personal protective equipment	Stethoscopes, goggles
Uniforms and accessories	Scrubs, white coats, badges
Writing implements	Pens, clipboards, markers
Personal devices	Phones, tablets, watches
Break room items	Microwaves, coffee makers
Storage items	Lockers, personal belongings
Educational materials	Textbooks, reference cards
*Common areas*	
Reception areas	Counters, sign-in materials
Waiting rooms	Furniture, magazines, toys
Public restrooms	All surfaces and fixtures
Hallways	Handrails, water fountains
Elevators	Buttons, rails, walls
Cafeteria/food service areas	Tables, chairs, trays
Vending machines and ice dispensers	
*Specialized areas*	
Medication rooms	Counters, refrigerators, cabinets
Treatment rooms procedure	Tables, equipment
Clean utility rooms	Supply storage, preparation areas
Dirty utility rooms	Waste containers, cleaning equipment
Isolation rooms	All surfaces require special attention
Operating rooms	All equipment and surfaces
Laboratory areas	Work surfaces, equipment
*Transportation equipment*	
Stretchers and transport beds	
Wheelchairs and accessories	
Patient transport vehicles	
Equipment carts	
Specimen transport containers	
Supply delivery carts	
Waste collection containers	
Drainage systems and water infrastructure	Sink siphons, drain traps, shower heads
Other	Visitors, food
*Public spaces surface prone to microbial contamination*	
Transportation	Bus/train seats, handrails, ticket machines, seat belts, airplane tray tables
Restaurants	Menus, tables, chairs, condiment bottles, salt/pepper shakers
Gyms/fitness centers	Treadmill handles, weights, yoga mats, locker room benches, shower floors
Schools/offices	Desks, chairs, whiteboard markers, shared computers, water coolers
Retail stores	Shopping carts, checkout counters, fitting room handles, product testers
Entertainment venues	Movie theater seats, arcade games, bowling balls, concert railings
Public restrooms	Toilet seats, faucets, door handles, paper towel dispensers, hand dryers
Parks/playgrounds	Swing sets, slides, picnic tables, water fountains, park benches
Hotels	Room keys, elevator buttons, remote controls, bed linens, bathroom fixtures
Libraries	Book covers, computer keyboards, study tables, magazine racks
Healthcare facilities	Waiting room chairs, reception counters, vending machines, handrails
Airports	Security bins, check-in kiosks, boarding pass scanners, luggage carts
Public events	Ticket booths, food stalls, portable toilets, seating areas
Public transit hubs	Escalator handrails, ticket counters, waiting area chairs, information kiosks
Grocery stores	Produce scales, freezer handles, self-checkout screens, basket handles

Adapted from references Kramer et al., 2024; Kramer et al., 2006; Porter et al., 2024; Stephens et al., 2019; Wißmann et al., 2021; Jabłońska-Trypuć et al., 2022.

### 2.1 Human and pets’ origins in indoor environments

The sources of inanimate surfaces microbial contamination are predominantly from humans, originating from skin, infected body fluids, including saliva, excreta (i.e., urine and feces), vomit, blood, nasal secretions, and respiratory secretions (respiratory droplets and droplet nuclei or aerosols; Boone and Gerba, 2007; Atmar et al., 2008; Lopez et al., 2013; Stephens et al., 2019). Studies have consistently shown that the microbiota of skin shedding dominate surfaces in spaces with high human occupancy and frequent interactions (Mackintosh, 1983; Sahay et al., 2018; Stephens et al., 2019). These microbial communities demonstrate remarkable adaptability, rapidly converging with the microbiota of new residents following relocation (Adams et al., 2015). In addition, empirical investigations indicated that droplets originating from humans can disseminate up to 1.25 meters during speech and 1.37 meters during coughing, facilitating microbial dispersion (Reyes et al., 2022). Coughing produces roughly 3,000 droplets, many of which are sufficiently large to settle quickly onto inanimate surfaces (Atkinson et al., 2009; Dhand and Li, 2020). Sneezing, in contrast, may generate as many as 40,000 droplets ranging from 0.5 to 12 μm in diameter, expelled at velocities approaching 100 m/s (Cole and Cook, 1998; Tang et al., 2006; Atkinson et al., 2009). Such forceful emissions dramatically increase the likelihood of droplet sedimentation. These microbial contaminants are deposited onto fomites through direct contact or indirect transfer via, e.g., contaminated hands, serving as critical vectors and intermediaries that transfer pathogens directly or indirectly to inanimate surfaces (Ansari et al., 1988; Lopez et al., 2013; Porter et al., 2024).

Microorganisms are systematically transferred from contaminated hands, with particular emphasis on finger pads, facilitating the spread of microorganisms including pathogens across inanimate surfaces (Ansari et al., 1988; Atmar et al., 2008; Lopez et al., 2013; Weber et al., 2013a,b; Bailey et al., 2019; Reyes et al., 2022). Numerous studies have comprehensively examined hand hygiene and pathogen transmission dynamics, especially within healthcare settings and at high-touch environmental interfaces (Ansari et al., 1988; Mbithi et al., 1992; Jabłońska-Trypuć et al., 2022). In hospital settings, healthcare workers’ hands can become contaminated after direct patient contact or after touching inanimate surfaces in the patient zone (Longtin et al., 2011; Jinadatha et al., 2017; Bhalla et al., 2021). Studies showed that 42–53% of healthcare workers’ hand cultures test positive for potentially pathogenic microorganisms after brief surface interactions (Longtin et al., 2011; Jinadatha et al., 2017; Bhalla et al., 2021). This process is complicated further by the involvement of mobile vectors, including healthcare workers’ phones, portable computers, medical devices, and clothing, which can act as secondary reservoirs for nosocomial pathogens (Bhoonderowa et al., 2014).

Among these mobile vectors, personal electronic devices such as mobile phones and tablets warrant particular attention due to their widespread use and high contamination rates. Personal electronic devices such as mobile phones and tablets are increasingly recognized as reservoirs of microbial contamination (Ahmed et al., 2025a; Ahmed et al., 2025b). Their frequent handling and proximity to the face facilitate bacterial transfer from skin, food, and respiratory secretions (Kuriyama et al., 2021). Surveys in large urban populations have shown that nearly half of mobile phones carry bacterial contamination, and more than one-third harbor potentially pathogenic organisms at clinically concerning levels (Cicciarella Modica et al., 2020). This is particularly alarming given that individuals outside healthcare settings often lack awareness of equipment hygiene compared to healthcare workers, increasing the likelihood of cross-contamination (Haque et al., 2018). The presence of pathogens such as *Staphylococcus aureus*, *Escherichia coli*, and *Pseudomonas aeruginosa* on these devices underscores their role as vectors of infection, capable of spreading illness in both community and hospital environments (Ahmed et al., 2025a; Ahmed et al., 2025b).

Certain microorganisms, such as *Staphylococcus aureus*, can persist not only on inanimate surfaces but also within human skin flora and mucosal sites (Kramer et al., 2006; Taylor et al., 2025). Epidemiological studies demonstrate that colonization with *Staphylococcus aureus* follows a heterogeneous pattern: approximately 20–30% of individuals are persistently colonized, most often in the anterior nares but also at other body sites (Howden et al., 2023; Taylor et al., 2025). Around 30% experience intermittent colonization influenced by host and environmental factors, while the remaining 40% are generally non-carriers (Touaitia et al., 2025), though transient colonization can occur under favorable conditions (Piewngam and Otto, 2024). This distribution highlights the role of asymptomatic carriers as reservoirs for recurrent infections, particularly in vulnerable populations. Importantly, *S. aureus* is easily transmitted through direct contact with animals, people, and inanimate objects, making both colonized individuals and contaminated surfaces critical drivers of its dissemination in community and healthcare settings alike (Howden et al., 2023; Taylor et al., 2025). Approximately 20–30% of individuals are persistent nasal carriers of *S. aureus*, and an additional 30% carry it intermittently. However, certain high-risk populations tend to exhibit significantly higher colonization rates, reaching up to 80%, including healthcare workers, people who use drugs (PWUDs), such as crack, cocaine, heroin (Gwizdala et al., 2011), people with diabetes, hospitalized patients, and immunocompromised individuals (Kramer et al., 2006; Howden et al., 2023). Beyond simple carriage, colonization by *S. aureus* constitutes a recognized risk factor for subsequent infection, particularly in immunocompromised individuals, surgical patients, and people with chronic conditions. Host susceptibility to colonization and infections depends on the immune system, the existence of chronic diseases, environmental conditions, and hygiene practices. The immune system may be compromised by prior exposure to pathogens, nutritional status, underlying diseases, pregnancy, immunosuppressive drugs, and malignancies (Boone and Gerba, 2007; Casadevall and Pirofski, 2018). Aging, in particular, is associated with immunosenescence, a decline in innate and adaptive immunity, which increases susceptibility to infections, particularly among older adults who often have additional comorbidities and may be on immunosuppressive medications (Casadevall and Pirofski, 2018; Theodorakis et al., 2024). Importantly, evidence from intensive care units demonstrates that enhanced environmental cleaning significantly reduces colonization and infection rates of multidrug-resistant bacteria (Li et al., 2021), highlighting the role of targeted interventions in mitigating host vulnerability.

In healthcare environments, these transmission complexities are especially evident (Taylor et al., 2025). The primary transmission cycle initiates when colonized or infected patients contaminate their immediate environment through skin, fluids, and respiratory droplets, particularly affecting high-touch surfaces such as bed rails, IV poles, monitoring equipment, and toilets (Bonten et al., 1996; Huslage et al., 2010). Healthcare workers subsequently serve as intermediaries in this transmission chain, potentially contaminating their hands and protective equipment during patient care activities or interaction with contaminated surfaces, such as portable equipment and mobile devices (Longtin et al., 2011; Bhoonderowa et al., 2014; Jinadatha et al., 2017; Bhalla et al., 2021).

Contaminated surfaces generally harbor lower pathogen concentrations (1–100 CFUs per cm^2^) than colonized patients (Bonten et al., 1996; Julian et al., 2010; Bhoonderowa et al., 2014). However, the risk remains significant because certain nosocomial pathogens have low infectious doses, making even minimal contamination clinically relevant (Kramer et al., 2024). By contrast, pathogens such as *Staphylococcus aureus* require much higher inocula (>10^5^–10^10^ CFU/mL) to establish infection, yet their persistence on surfaces and repeated opportunities for transfer still sustain transmission risks (Duckro et al., 2005; Julian et al., 2010; Kramer et al., 2024)., Genomic studies have revealed cross-contamination pathways with shared medical equipment serving as mobile reservoirs and facilitating pathogen spread between different hospital areas (Hayden et al., 2008). Numerous studies, including systematic reviews, demonstrated a strong correlation between increased pathogen acquisition risks in rooms previously occupied by infected or colonized patients (Huang et al., 2006; Agodi et al., 2007; Nseir et al., 2011; Mitchell et al., 2015; Mitchell et al., 2023). The documented pathogens of concern included Methicillin-resistant *S. aureus* (MRSA), vancomycin-resistant *enterococci* (VRE), *Klebsiella* spp., extended-spectrum *β*-lactamase (ESBL)-producing *Escherichia coli*, *Clostridium difficile*, multidrug-resistant *Pseudomonas aeruginosa, Acinetobacter baumannii*, and norovirus (Huang et al., 2006; Agodi et al., 2007; Nseir et al., 2011; Mitchell et al., 2015; Mitchell et al., 2023).

Microbial transmission pathways extend to community areas. High-touch shared surfaces in public areas such as doorknobs, countertops, and personnel belongings, serve as critical fomites for microbial contamination and transmission (Stephens et al., 2019). Public transport systems represent critical hubs of microbial transfer, with high passenger density and frequent contact with shared surfaces such as door buttons, handrails, and ticket machines (Ly et al., 2024). The widespread contamination risk is particularly significant as these public spaces intersect with healthcare environments, potentially contributing to the broader circulation of pathogens within communities (Kramer et al., 2006). Studies have demonstrated the presence of clinically significant pathogens in public transportation systems (Simoes et al., 2011; Conceicao et al., 2013). MRSA was detected on bus handrails and passengers’ hands (Simoes et al., 2011; Conceicao et al., 2013). The survival of these microorganisms on inanimate surfaces is affected by multiple environmental and surface-specific factors (Katzenberger et al., 2021; Jabłońska-Trypuć et al., 2022). The interconnected nature of these contamination pathways extends beyond human-centric environments, with documented pathogen transfer between animals and inanimate objects, creating potential routes for zoonotic transmission (Gentile et al., 2020).

Pets can be an important source of zoonotic disease transmission, including resistant pathogens (Stull et al., 2015). Transmission occurs through multiple routes: direct contact with saliva, urine, feces, or scratches; ingestion of contaminated food or water; indirect exposure via insect vectors or contaminated objects; and inhalation of infectious aerosols (Udainiya et al., 2024). Documented zoonoses include campylobacteriosis, leptospirosis, staphylococcal infections, salmonellosis, *E. coli* infections, and psittacosis (Ghasemzadeh and Namazi, 2015; Stull et al., 2015). Although surveys and epidemiologic studies suggest that the overall occurrence of pet associated disease is low, the true burden is likely underestimated owing to under reporting, non pet exposure pathways, and frequent subclinical shedding. Companion animals have been implicated in more than 70 human diseases, with young children, older adults, pregnant women, and immunocompromised patients at greatest risk (Stull et al., 2015). Some infections may be life threatening: cats and dogs can transmit Bartonella species, which in immunocompromised or asplenic patients may cause severe systemic illness such as bacteremia, endocarditis, neuroretinitis, or bacillary angiomatosis (Álvarez-Fernández et al., 2018). Pets may also harbor multidrug resistant organisms including MRSA and *Clostridium difficile* (Morris et al., 2012), further intensifying public health concerns. These examples highlight that while pets provide important health and social benefits, they can also act as reservoirs of clinically significant and resistant pathogens, underscoring the need for awareness and preventive measures in vulnerable households.

### 2.2 Environmental origins of microbial contamination

Environmental contamination is defined as the introduction of hazardous agents into air, water, soil, or surfaces at levels harmful to human health or ecosystems, primarily driven by human activities (Imran, 2023). Within healthcare and community settings, microbial contamination represents a key dimension of this phenomenon. Broad determinants such as climate, hygiene, crowding, and exposure to toxins shape host–microbe interactions and infection risk (Kumar et al., 2021). Beyond cleaning and disinfection, the design and material characteristics of the built environment serve as critical barriers to interrupt transmission, providing a structural foundation for infection prevention.

#### 2.2.1 Air, dust, and human activities

Microbial contamination of indoor surfaces results from a complex interplay between human occupants, environmental factors, and microbial ecology.

In healthcare settings, the spread of MDROs through environmental contamination represents a critical epidemiological challenge, particularly the sequential room occupancy patterns (Nseir et al., 2011; Mitchell et al., 2015; Porter et al., 2024). While the precise molecular mechanisms underlying pathogen acquisition remain incompletely characterized, compelling evidence supports environmental contamination as a pivotal transmission vector, despite inherent limitations in establishing direct causality through surface sampling methods, and genomic or cultural analysis (Nseir et al., 2011; Mitchell et al., 2015). Rigorous empirical investigations, primarily conducted within ICUs, have consistently demonstrated persistent microbial contamination following terminal cleaning protocols, even with strict adherence to standardized cleaning procedures (Boyce, 2007; Russotto et al., 2015).

In household settings, surfaces in indoor environments are subject to contamination through airborne deposition of biological particles (Dunn et al., 2013). Multiple reservoirs and transmission vectors facilitate microbial persistence in household environments. For example, in areas of frequent contact, such as floors, microbial compositions closely mirror those found on human feet (Adams et al., 2015). House dust serves as a primary reservoir, with organisms like *Mycobacterium abscessus* showing enhanced survival and growth when associated with particulate matter and environmental matrices such as kaolin (González-Martín et al., 2021). Aerosol-mediated transmission adds another layer of complexity to the dynamics of microbial transmission in household settings. Bioaerosols, including bacteria, fungi, viruses, pollen, and microbial metabolites, account for 5–34% of indoor air pollutants (González-Martín et al., 2021; Chen et al., 2024), with particle sizes usually ranging from 0.001 nm to 100 μm (Al Hallak et al., 2023). Dominant airborne microorganisms show consistency between indoor and outdoor environments (Kramer et al., 2006; Chen et al., 2024; Kramer et al., 2024). They include *Anoxybacillus, Brevibacillus,* and *Acinetobacter* as prevalent bacteria, and *Cladosporium, Penicillium,* and *Alternaria* as prevalent fungi (Al Hallak et al., 2023; Chen et al., 2024). Seasonal variation in indoor microbial concentrations has been documented in several countries, though the specific dynamics differ by climate and context (Al Hallak et al., 2023; Chen et al., 2024; Mengyao et al., 2025). In Denmark, fungal concentrations peak in summer while bacterial loads are highest in spring, whereas in the United States, peak bacterial concentrations in office environments occur variably in summer or winter (Mengyao et al., 2025). In China, airborne microbiomes show higher abundance in summer, with indoor bioaerosols largely originating from outdoor sources (Chen et al., 2024). Seasonal patterns significantly influence these dynamics, with microbial abundance generally highest in summer and indoor bioaerosols primarily linked to outdoor sources, while temperature, relative humidity, and particulate matter concentrations correlate with airborne microbiome levels and subsequent surface deposition rates (Al Hallak et al., 2023; Chen et al., 2024). These findings indicate that season alone is not a decisive factor, as human behaviors (e.g., crowding in confined spaces during colder months) and environmental conditions jointly shape microbial dynamics across settings.

Particulate matter, including PM2.5 and PM10, serves as vectors and protective carriers for both resistant bacteria and resistance genes in air dust, allowing them to travel across distances and deposit pathogens onto inanimate surfaces in healthcare and community settings (Zhou et al., 2023; Sassi et al., 2025). Importantly, resistance determinants detected in particulate matter may exist either within viable airborne microorganisms or as extracellular DNA fragments bound to particulates. Current molecular detection methods (e.g., qPCR, metagenomics) cannot fully distinguish between these forms, but both represent potential reservoirs that contribute to the dissemination of antimicrobial resistance (Sassi et al., 2025).

Pathogenic microorganisms persist in hospital indoor air environments, with recovery rates sometimes exceeding 92.5% from air samples, indicating that many pathogenic microbes remain suspended and subsequently deposit onto inanimate surfaces (Leta et al., 2014; Solomon et al., 2017; Belay et al., 2024). The predominant airborne organisms include *S. aureus*, coagulase-negative *staphylococci*, *Aspergillus* spp., and GNB (Leta et al., 2014; Solomon et al., 2017; Belay et al., 2024).

Appropriate and effective operation of heating, ventilation, and air conditioning (HVAC) systems is a priority for improving hospital indoor air quality (Saran et al., 2020; Xu and Zhou, 2016; Nikoopayan Tak and Mousavi, 2025), and high-efficiency particulate air (HEPA) filters are recommended for infection control in high-risk areas (Landry et al., 2022; Arıkan et al., 2022). These technologies can efficiently filter aerosol particles regardless of their biogenic origin, and numerous studies have reported that HEPA filters reduce the risk of *aspergillus* spp. and other fungal infections (Eckmanns et al., 2006). HVAC-integrated air filtration systems, including indoor air purifiers, have also been shown to decrease aerosolized viral loads, and HEPA filters are particularly effective in reducing airborne levels of SARS-CoV-2, the causative agent of COVID-19 (Liu et al., 2022). Reinforcing existing ventilation systems with environmental and administrative controls therefore remains a cornerstone strategy for minimizing hospital-acquired infections associated with airborne pathogens (Arıkan et al., 2022).

Higher microbial loads in indoor air are associated with increased human traffic, crowding, and aerosolization of dust particles (Belay et al., 2024). In hospital settings, morning hours show peak contamination due to medical rounds and patient activities (Belay et al., 2024). Antimicrobial-resistant bacteria (ARB) in air demonstrate survival periods of 8 to 72 h, facilitated by protective organic coatings that enable airborne dispersal and subsequent deposition onto environmental surfaces (Jones and Brosseau, 2015). Given the persistence of pathogens on surfaces and their potential for airborne transmission (Kramer et al., 2006; Boyce, 2007; Russotto et al., 2015; Stephens et al., 2019; Wißmann et al., 2021; Katzenberger et al., 2021; Jabłońska-Trypuć et al., 2022; Reyes et al., 2022; Kramer et al., 2024; Porter et al., 2024), the implementation of air treatment systems, particularly high-efficiency filtration technologies, is highly relevant in high-risk areas such as hospital wards and also in industrial environments, as supported by recent reviews and studies on indoor air control (Leta et al., 2014; González-Martín et al., 2021; Al Hallak et al., 2023; Chen et al., 2024; Belay et al., 2024; Sassi et al., 2025). Additionally, building interfaces with the outdoor environment, such as interior and exterior trim, harbors distinct microbial communities predominantly associated with environmental sources like leaves and soil (Lax et al., 2017; Stephens et al., 2019).

#### 2.2.2 Water drainage systems

Water drainage systems, including sinks, siphons, handwashing basins, shower drains, potable water systems, and associated plumbing infrastructure, represent a critical source of inanimate surface contamination across household, hospital, and community settings (Williams et al., 2013; Li et al., 2023; Hassan et al., 2025; CDC, 2024; Hayward et al., 2025; Hennebique et al., 2025). These drainage systems serve as persistent inanimate reservoirs where stagnant water facilitates the development of complex microbial biofilm communities of bacteria, fungi, and protozoa resistant to antimicrobial agents and disinfection interventions (Li et al., 2023; Sharma et al., 2023). These waterborne environments serve as distinct ecological pathways where biofilm facilitates the spread of resistance under adverse conditions and persistent contamination (Hassan et al., 2025). Biofilms frequently develop on surfaces such as walls, sinks, shower curtains, and countertops, with community compositions reflecting those found in associated plumbing systems and water reservoirs (Hussain et al., 2024; Putri et al., 2025). These biofilms can serve as persistent sources of contamination and potential transmission (Chen et al., 2024; Ban-Cucerzan et al., 2025). Hospital wastewater plumbing systems, particularly patient-room sinks, represent ideal reservoirs where environmental and pathogenic bacteria interface and exchange antimicrobial-resistance genes (McCallum and Hall, 2025; Kotay et al., 2026). Hospital sink drains can become “melting pots” for antimicrobial resistance, where exposure to disinfectants, medications, other nutrients such as leftover drinks, and body fluids fosters horizontal gene transfer of resistance genes to nosocomial pathogens (Ward et al., 1991; McCallum and Hall, 2025). Recent metagenomic analyses of intensive-care unit sink drains demonstrated that plumbing replacement, although widely deployed as an intervention, can paradoxically increase the abundance of Enterobacterales and antimicrobial-resistance genes, as disruption of established biofilms destabilizes microbial communities and promotes recolonization by resistant taxa (Kotay et al., 2017; De Geyter et al., 2017; Kotay et al., 2026). Exposure to these nutrients can enrich drain biofilms and sustain microbial growth and resilience (Kotay et al., 2017).

Such practices increase the risk of pathogen dispersal through splash-out and aerosolization.

dispersing pathogenic microorganisms onto surrounding surfaces and exposing individuals in different settings to waterborne pathogens (Centers for Disease Control and Prevention, 2024; Kotay et al., 2017; Dieter et al., 2025). This risk is particularly well-documented in patients with cystic fibrosis (CS), for whom enhanced vigilance regarding the hygiene of water drainage systems is strongly recommended (Schelstraete et al., 2008; Saiman et al., 2014; Purdy-Gibson et al., 2015). Due to their chronic lung disease and long-term exposure to broad-spectrum antibiotics, CF patients are highly susceptible to colonization by opportunistic waterborne pathogens, most notably *P. aeruginosa*, which is frequently recovered from kitchen, sink drains, shower drains, and plumbing biofilms in home and clinical environments (Schelstraete et al., 2008; Saiman et al., 2014; Purdy-Gibson et al., 2015).

Activities such as toilet flushing can generate bioaerosols containing microorganisms from excreta and vomit, producing thousands of particles per event. Daily activities such as sweeping and vacuuming contribute to bioaerosol resuspension from surfaces (Al Hallak et al., 2023). These aerosols can remain suspended before settling on surfaces, contributing to fomite-mediated contamination (Al Hallak et al., 2023). During both symptomatic and asymptomatic infections, these emissions can release millions of pathogens, highlighting their significance in disease transmission (Goldman, 2000; Zhao et al., 2005; Johnson et al., 2017). Beyond these moisture-rich niches, recent research has highlighted the clinical and industrial significance of ‘dry biofilms’ (DSB) (Almatroudi et al., 2018; Centeleghe, 2022; Ledwoch et al., 2022; Schapira et al., 2024; De-la-Pinta et al., 2026). Unlike their hydrated counterparts, dry biofilms exhibit a unique architecture and physiology (Almatroudi et al., 2018; Centeleghe, 2022; Ledwoch et al., 2022; Schapira et al., 2024; De-la-Pinta et al., 2026). They are often compact, highly adherent, and can enter a dominant viable but non-culturable (VBNC) state, allowing them to persist for extended periods (weeks to months) even without a defined nutrient source. Importantly, cells within dry biofilms demonstrate a markedly increased tolerance to standard disinfectants and cleaning protocols, posing a significant challenge for decontamination and representing a persistent, often undetected, reservoir for potential transmission (Almatroudi et al., 2018; Centeleghe, 2022; Ledwoch et al., 2022; Schapira et al., 2024; De-la-Pinta et al., 2026).

In healthcare settings, water drainage systems represent a critical secondary source of inanimate surface contamination. Contaminated sinks have been directly implicated in outbreaks of carbapenem-resistant *P. aeruginosa* and Enterobacteriaceae, often persisting despite cleaning, disinfection procedures, and structural interventions such as siphon or sink replacement (Hassan et al., 2025). According to the Centers for Disease Control and Prevention (CDC, 2024) considerations for reducing risk related to water in healthcare facilities, opportunistic pathogens of premise plumbing include a wide range of microorganisms. GNB represents the predominant category, in addition to nontuberculous mycobacteria, most notably the *M. abscessus* clade, *M. avium* complex, and *M. fortuitum* clade (CDC, 2024). Fungal pathogens encompass yeasts such as *Candida* spp. and molds, including *Aspergillus, Fusarium,* and *Exophiala* species. Additionally, protozoan parasites, including *Acanthamoeba, Vermamoeba vermiformis*, and *Naegleria* species, can inhabit these water systems and expose to health risks (CDC, 2024). A study investigating sink drain biofilms in hospital intensive care units demonstrated that ARGs and mobile genetic elements show no significant differences in abundance between drains receiving routine disinfection and those without routine disinfection protocols (Hennebique et al., 2025). Despite distinct bacterial community compositions between the two units, the resistome profiles remained equivalent, indicating that ARG abundance is independent of microbiota composition and disinfection practices. This finding highlights that drainage biofilms are persistent reservoirs of AMR in healthcare environments (Hennebique et al., 2025).

#### 2.2.3 Manufactured products, materials and equipment

Post-marketing contamination represents a critical regulatory gap between manufacturing controls and personal use. Studies found that contamination rates of used medicines, medical products, personal care products, and cosmetics range from 2 to 100% despite quality control and stringent regulatory oversight (Roy et al., 2023; Osuoha et al., 2023; Gupta et al., 2024; Tyski et al., 2025; da Silva et al., 2025). The predominant microbial contaminants include GPB such as *S. aureus*, and GNB, like *P. aeruginosa*, *Enterobacter* spp., in addition to fungi and molds (Jarvis et al., 2014; Alhaddad et al., 2015; Peckham et al., 2016; Riquena et al., 2019; Haston, 2023; Navadifar et al., 2023). Biofilm formation is found across home-used nebulizers, contact lens cases, and breastfeeding equipment, despite reported compliance with manufacturer instructions for hygiene maintenance. Immunocompromised individuals, neonates, and elderly persons are at high risk of associated infection, including keratitis, respiratory exacerbations, and neonatal sepsis (Jarvis et al., 2014; Alhaddad et al., 2015; Peckham et al., 2016; Riquena et al., 2019; Haston, 2023; Navadifar et al., 2023).

In healthcare settings, microbial contamination of medicines, medical equipment, and materials is a leading cause for colonization, nosocomial infections, and disease complications among hospitalized patients (Russotto et al., 2015; Saka et al., 2017; Gruszecka et al., 2019; Ndu et al., 2022; Prasek et al., 2025). They may be heavily contaminated with pathogens, including MDR bacteria (Russotto et al., 2015; Saka et al., 2017; Gruszecka et al., 2019; Ndu et al., 2022; Prasek et al., 2025). A systematic review indicated that the rate of microbial contamination of medical equipment can reach 86.8% if cleaning and disinfection protocols are not strictly applied (Schabrun and Chipchase, 2006).

#### 2.2.4 Kitchen surfaces and food

The domestic kitchen environment presents particularly significant contamination risks due to its role in food preparation. Common foodborne pathogens, including *Listeria monocytogenes*, *S. aureus*, *Salmonella* spp., and *Campylobacter jejuni*, frequently colonize kitchen surfaces and utensils (Castano et al., 2021). Contamination pathways are diverse: raw meat and poultry may introduce pathogens from processing-related exposure to intestinal contents, while fresh produce can harbor microorganisms from contaminated irrigation sources or soil (Evans and Redmond, 2019; Kuan et al., 2020; Link et al., 2021). Beyond their role as vectors introducing pathogens, contaminated foods themselves constitute an ecological niche conducive to microbial proliferation in the kitchen (Baroni et al., 2013; Link et al., 2021; Evans and Redmond, 2019; Thames and Theradiyil Sukumaran, 2020; Brown et al., 2014; Chen et al., 2018). Food residues on countertops, cutting boards, or in the sink provide essential nutrients, such as water, carbohydrates, and proteins that enable the survival and growth of microorganisms, including those introduced by the contaminated food itself (Baroni et al., 2013; Link et al., 2021; Evans and Redmond, 2019; Thames and Theradiyil Sukumaran, 2020; Brown et al., 2014; Chen et al., 2018). This microbial amplification on surfaces, if not controlled by adequate cleaning and disinfection, increases the risk of cross-contamination to other foods and kitchen surfaces (Baroni et al., 2013; Link et al., 2021; Evans and Redmond, 2019; Thames and Theradiyil Sukumaran, 2020; Brown et al., 2014; Chen et al., 2018). For example, a spill of raw poultry juice on a countertop represents not only an immediate introduction of *Campylobacter* spp. or *Salmonella* spp., but also creates a moist, nutrient-rich micro-environment that, at room temperature, can allow these bacteria to multiply, thereby amplifying the hazard (Brown et al., 2014; Chen et al., 2018).

## 3 Transmission pathways between inanimate surfaces and humans

**Figure 2:**
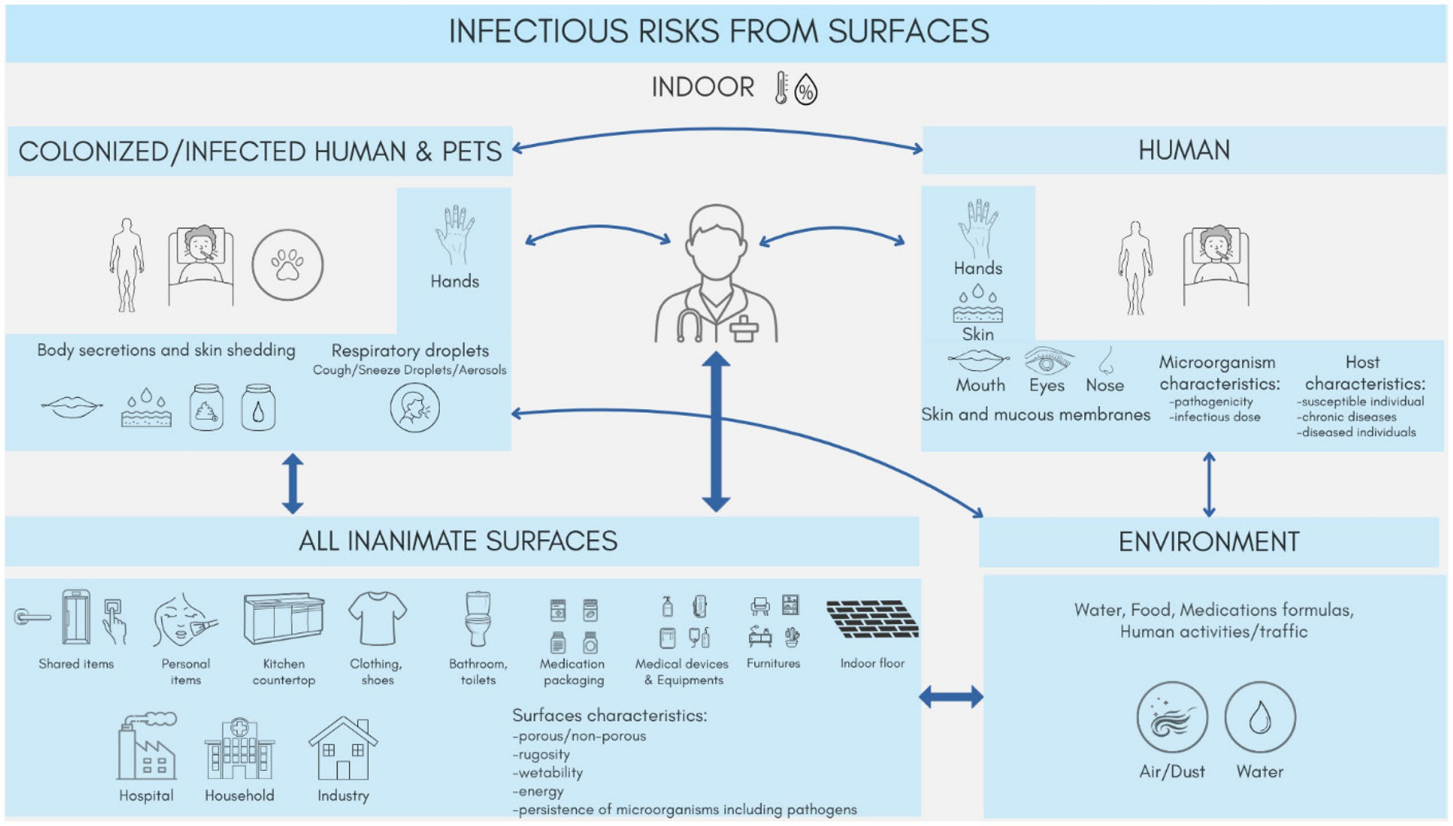
Infectious risk pathways from inanimate surfaces. Original conceptual figure created by the authors using Canva. The schematic describes the interconnected pathways through which infectious agents spread indoors. Colonized or infected humans and pets shed microorganisms through respiratory droplets, aerosols, secretions, and skin. These contaminants are transferred directly between individuals (healthy, susceptible, colonized, diseased) or indirectly via hands, clothing, and onto inanimate surfaces, spreading in the environment. Healthcare providers can act as intermediaries, acquiring pathogens from patients or contaminated surfaces and transmitting them onward. Environmental reservoirs (air, dust, and water) amplify circulation, spreading microbial contamination back onto inanimate surfaces and hosts. Arrows indicate bilateral pathways: surfaces, individuals, and the environment reciprocally exchange microbial contamination. Thick arrows indicate infectious risks originating from inanimate surfaces.

The multi-directional pathways of microbial contamination from inanimate surfaces involve a complex cycle of infection transmission with multiple interconnected routes across healthcare, household, and industrial settings, highlighting the dynamic nature and sources of surface -mediated disease spread (Figure 2). Building on this framework, Section 3 examines the environmental and surface parameters that influence pathogen persistence, survival, and infection risk.

### 3.1 Microorganisms’ parameters

The ability of a microorganism to cause infection is governed by a set of intrinsic microbial characteristics that collectively define its pathogenic potential. Understanding these characteristics is a mainstay in assessing the risk of microbial transmission in community and healthcare settings.

#### 3.1.1 Persistence and replication capacity

The persistence of pathogenic microorganisms on inanimate surfaces, defined as their ability to survive over extended periods, represents a fundamental determinant of their transmission potential and epidemiological significance (Kramer et al., 2006). This temporal survival capacity is necessary but often insufficient for replication (Kramer et al., 2024). Persistence is crucial in microbial transmission through contaminated environmental surfaces (Boone and Gerba, 2007). It manifests through complex adaptive mechanisms that enable pathogens to maintain viability under adverse environmental conditions, including metabolic reduction, morphological transformations, adhesion, biofilm formation, and transition to VBNC states and spores (Katzenberger et al., 2021; Kramer et al., 2024). Persistence is a prerequisite for pathogen transmission modulated by a complex interplay of factors, including environmental parameters, microorganism-specific attributes, and surface characteristics (Porter et al., 2024).

The distinction between persistence and replication capacity (RC) represents a crucial evolution in understanding pathogen behavior on inanimate surfaces, with each concept offering unique insights while exhibiting complementary strengths and limitations (Kramer et al., 2024). Kramer et al. (2024) highlighted that the advancements in RC assessment led to more precise measurements based on inoculum calculations and log reduction values, providing superior data compared to earlier persistence studies. RC addresses a pathogen’s ability to replicate and cause infection after surface contamination, making it particularly relevant for clinical risk assessment (Kramer et al., 2024). Modern research using whole-genome sequencing has revealed that standard techniques significantly underestimate transmission events (Ledwoch et al., 2018; Ledwoch et al., 2022; Centeleghe, 2022), indicating the importance of persistence and RC measurements. These concepts share common biases, particularly in laboratory studies that often represent worst-case scenarios and may overestimate actual transmission risks (Wißmann et al., 2021; Kramer et al., 2024; Porter et al., 2024). Environmental factors such as temperature, humidity, and surface materials affect persistence and RC (Kramer et al., 2024; Jabłońska-Trypuć et al., 2022). The relationship between persistence and RC is complicated when considering collective infectious units, where multiple pathogens can aggregate in lipid vesicles or protein matrices (Sanjuan and Thoulouze, 2019; Kramer et al., 2024), potentially enhancing their persistence and replication potential. Despite their limitations, integrating persistence and RC data provides a more comprehensive understanding of pathogen transmission through inanimate surfaces, leading to novel classification systems based on surface-borne transmission potential rather than traditional taxonomic approaches (Kramer et al., 2024). These systems require further scientific validation while considering patient safety, employee protection, and ecological sustainability in infection control strategies.

#### 3.1.2 Minimum infectious dose and pathogenicity

The minimum Infectious Dose (MID) represents a critical microbial characteristic in the transmission risk from contaminated inanimate surfaces (Seyed Alinaghi et al., 2022; Kramer et al., 2024). Its determination requires careful distinction between *in vitro* surrogates and actual *in vivo* infectious potential. Laboratory viral quantifications, typically employing tissue culture infectious dose (TCID₅₀) and plaque-forming units (PFU) (Ward et al., 1984; Sanjuan and Thoulouze, 2019; Kramer et al., 2024), serve as correlates but do not directly equate to the infectious dose in living hosts. Rather, the true MID is inherently correlated to the pathogenicity of the microorganism and is modulated by complex host-pathogen interactions that cannot be fully recapitulated in cell culture systems (Yezli and Otter, 2012; Kramer et al., 2024).

Kramer et al. (2024) highlights that transmission via surface-to-hand-to-mucosa routes presents an infection risk when pathogens exhibit low MIDs (Kramer et al., 2024). Direct contact with contaminated surfaces followed by inoculation of the mouth, nose, or eye can initiate infection (Kramer et al., 2024). Ocular exposure directly from surfaces or indirectly via hands represents a particular concern for pathogens causing epidemic keratoconjunctivitis (Kramer et al., 2024). Conversely, for respiratory viruses requiring more than 10^2^ TCID₅₀ to establish infection, aerosolization from surfaces is considered an unlikely transmission pathway. Similarly, fecal-orally transmitted bacteria with MIDs below 10^2^ CFU are not typically associated with surface-mediated transmission (Kramer et al., 2024).

However, for pathogens where surface transmission does occur, the published literature documents remarkably low infectious doses. Enteric viruses and bacteria, including norovirus, rotavirus, and *Clostridioides difficile*, can establish infection with as few as 1–100 particles (Yezli and Otter, 2012; Kramer et al., 2024). Respiratory viruses exhibit considerable variability, with rhinovirus requiring as little as 0.03 to >10^1^–10^4^ TCID₅₀ depending on serotype (Yezli and Otter, 2011). Kramer’s synthesis of MID data further illustrates this heterogeneity (Kramer et al., 2024): respiratory syncytial virus (RSV) requires 30–40 TCID₅₀ via intranasal inoculation (Yezli and Otter, 2012), while influenza A (H3N2) demands > 10^3^ TCID₅₀ through inhalation (Yezli and Otter, 2011). Influenza B demonstrates even higher requirements, ranging from 10^4^ to 10^7^ TCID₅₀ (Yezli and Otter, 2011). Adenovirus presents striking route-dependent variation, necessitating only 6.6 particles for inhalational exposure yet exceeding 10^6^ TCID₅₀ for oral inoculation (Yezli and Otter, 2011). Bacterial pathogens similarly display diverse infectious doses, with *Salmonella enteritidis* requiring ≥10^3^ CFU orally (Blaser and Newman, 1982), *P. aeruginosa* needing ≥10^4^ CFU via conjunctival exposure (Price and Ahearn, 1988), and commensal organisms such as *E. coli* and *S. aureus* requiring >10^5^ CFU for oral infection (Schmid-Hempel and Frank, 2007). Particularly, infection through the surface-finger-nose route for respiratory viruses may occur with a MID below 10^1^ TCID₅₀, as well as orally transmissible pathogens requiring fewer than 10^1^ TCID₅₀, CFU, or oocysts. In contrast, fecal-orally transmitted bacteria and Mucorales with MIDs below 10^2^ CFU are considered unlikely to spread via surface contact (Kramer et al., 2024). A critical limitation of conventional MID studies must be acknowledged: they typically fail to account for pathogen multiplication following initial acquisition. Microorganisms acquired in small numbers from contaminated surfaces may proliferate on or within the host, with clinical infection manifesting only after a critical burden has been reached (Kramer et al., 2024). Multiple factors influence the risk of infection from contaminated surfaces. These include the persistence of pathogens in various environments, their resistance to drying, and the individuals’ immune status. The risk is higher for vulnerable groups such as the elderly, newborns, and immunosuppressed patients. The route of exposure also matters, as pathogens infect differently through conjunctival, nasal, oral, or inhalational routes. Kramer et al. (2024) notes that when high environmental resistance combines with a low MID, using fast-acting disinfectants on surfaces is essential to control transmission (Kramer et al., 2024). This complexity is especially important in healthcare. Viral infections can increase the risk of developing secondary bacterial infections, lowering the amount needed to cause illness. Therefore, even low levels of surface contamination, below known MID values, can result in infection if the organism multiplies in a vulnerable patient.

#### 3.1.3 Microorganism-specific characteristics

Microorganism-specific characteristics play a crucial role in determining pathogen persistence on inanimate surfaces, with distinct patterns observed across different taxonomic groups (Otter et al., 2011; Thompson and Bennett, 2017; Wißmann et al., 2021; Jabłońska-Trypuć et al., 2022). Beyond taxonomic classification, several key structural and functional features influence microbial survival on surfaces. Surface hydrophobicity affects adhesion to abiotic and biotic surfaces (Krasowska and Sigler, 2014; Kreve and Dos Reis, 2021). More hydrophobic organisms generally exhibit enhanced attachment and subsequent persistence (Krasowska and Sigler, 2014; Kreve and Dos Reis, 2021). Bacterial appendages, including pili and fimbriae, facilitate initial surface colonization through specific and non-specific interactions, while adhesins (i.e., proteins located on the microbial surface) mediate targeted binding to host cells or extracellular matrix components deposited on surfaces (Kreve and Dos Reis, 2021). These adhesive properties, combined with variations in cell wall architecture (e.g., peptidoglycan thickness in GPB versus GNB), capsule production, and biofilm-forming capacity, collectively determine the extent to which a microorganism can establish and persist in contaminated environments (Wißmann et al., 2021; Jabłońska-Trypuć et al., 2022; Kramer et al., 2024). The following sections examine these characteristics across bacteria, viruses, and fungi.

##### 3.1.3.1 Bacteria

These characteristics vary considerably among bacteria. Flagella, when present, are implicated in the bacteria’s mobility and chemotaxis (Colin et al., 2021). Bacteria employ adhesins that interact with conditioned surfaces, enabling the formation of biofilms (Berne et al., 2015; Berne, 2023). Pili and adhesins contribute to surface colonization in species such as *K. pneumoniae* and *E. coli* (Schroll et al., 2010; Flores-Mireles et al., 2015; Sheikh et al., 2017).

Microorganism-specific attributes play a crucial role in determining pathogen persistence on inanimate surfaces, with distinct patterns observed across different taxonomic groups (Otter et al., 2011; Thompson and Bennett, 2017; Wißmann et al., 2021; Jabłońska-Trypuć et al., 2022). In general, GPB exhibit longer survival on dry surfaces than GNB due to their thicker peptidoglycan layer and greater resistance to desiccation (Jabłońska-Trypuć et al., 2022; Kramer et al., 2024). However, persistence times are highly dependent on environmental conditions such as temperature, relative humidity, organic load, and surface type. GNB and GPB exhibit variable persistence. For example, *K. pneumoniae* can survive for up to 600 days under specific optimal conditions (e.g., low temperature, high humidity, and the presence of organic soil), followed by *S. aureus* at 318 days under similar controlled conditions (Wißmann et al., 2021; Jabłońska-Trypuć et al., 2022). These extreme durations represent maximum reported persistence in experimental settings and do not reflect typical survival under real-world conditions. However, *Bacillus* and *Clostridium* species, particularly *C. difficile*, present exceptional contamination threats due to their ability to form highly resilient spores. While vegetative cells of *C. difficile* can survive up to 15 min on dry surfaces and 6 h on moist surfaces, spores are far more durable (Jump et al., 2007; Weber et al., 2013a,b; Malyshev et al., 2023). These spores persist for weeks to months on surfaces such as stainless steel and glass, and up to 5 months on flooring materials, withstanding desiccation, heat, and chemical disinfectants (Jump et al., 2007; Weber et al., 2013a,b; Malyshev et al., 2023). The spores’ multi-layered structure, particularly the dehydrated core, enhances resistance to environmental stressors. They adhere strongly to surfaces and remain stable during freezing and thawing cycles, acting as persistent reservoirs for transmission even with minimal inoculum (Otter and French, 2009; Broukhanski and Budylowski, 2019).

A critical factor contributing to the persistence of microorganisms on inanimate surfaces is their ability to form biofilms, a phenomenon extensively documented in healthcare settings (Vickery et al., 2012; Ledwoch et al., 2018; Jabłońska-Trypuć et al., 2022; Kramer et al., 2024). Biofilms represent sophisticated, structured heterogeneous microbial communities embedded within a self-produced extracellular polymeric matrix (EPS), demonstrating remarkable adaptability and resilience (Vickery et al., 2012; Ledwoch et al., 2018; Broukhanski and Budylowski, 2019; Kramer et al., 2024). Of particular clinical significance is their persistent survival on healthcare surfaces even after rigorous terminal cleaning and disinfection protocols (Vickery et al., 2012; Russotto et al., 2015; Ledwoch et al., 2018). These complex biological matrices substantially enhance pathogen survival within healthcare environments (Vickery et al., 2012). Compelling evidence demonstrate the viability of multidrug-resistant (MDR) bacteria, including *P. aeruginosa* and *A. baumannii*, isolated from biofilms persisting on clinical surfaces (Vickery et al., 2012). The biofilm phenotype confers unprecedented sensitivity loss to both physical and chemical decontamination strategies, with bacterial populations exhibiting up to 1,500-fold increased resistance to biocides compared to their planktonic counterparts (Fux et al., 2005; Russotto et al., 2015; Vickery et al., 2012). This remarkable enhancement in resistance potentially explains the persistent surface contamination observed despite the implementation of standardized terminal cleaning/disinfection protocols, thereby highlighting significant limitations in current methodological approaches (Vickery et al., 2012; United Nations, 2015; Stull et al., 2015). The formation of these sophisticated biofilm structures is triggered by various environmental stress conditions, ultimately leading to the development of a complex three-dimensional architecture (Vickery et al., 2012) that represents the predominant state of microbial life. The protective matrix comprises essential components, including water, proteins, lipids, polysaccharides, and extracellular DNA (Fux et al., 2005; Maillard and Denyer, 2009; Flemming and Wingender, 2010; Machado et al., 2012; Otter et al., 2015; Flemming et al., 2016; Shao et al., 2022; Kramer et al., 2024), collectively conferring robust protection against desiccation and exposure to various environmental stressors (Maillard and Denyer, 2009; Flemming and Wingender, 2010; Machado et al., 2012; Otter et al., 2015; Flemming and Wingender, 2010; Flemming et al., 2016; Jabłońska-Trypuć et al., 2022). Exopolysaccharides constitute a major structural component of this matrix, with their composition varying significantly between microbial species and substantially influencing the biofilm’s overall biomass, architectural organization, and functional capabilities. Within these densely packed microbial communities, microorganisms exhibit remarkable adaptability through the modification of their gene expression patterns and phenotypic characteristics via sophisticated quorum-sensing systems, enabling substantially enhanced adaptation to stressful environmental conditions compared to their free-floating planktonic counterparts (Flemming and Wingender, 2010).

##### 3.1.3.2 Viruses

Viral persistence varies significantly based on structural type. In general, viruses remain viable longer on non-porous materials (Vasickova et al., 2010). For example, non-enveloped DNA viruses, such as adenoviruses, can remain infectious on surfaces for days to several weeks, depending on environmental conditions and surface type, with longer survival reported on porous substrates under cooler temperatures (Abad et al., 1994; Boone and Gerba, 2007). Likewise, Abad et al. (1994) also reported greater persistence of rotavirus and poliovirus at high RH on non-porous materials. Building on these findings, Abad et al. (2001) found that astrovirus survival was longest at high relative humidity (90 ± 5%) and 4 °C, persisting for up to 60 days on non-porous surfaces and 90 days on porous substrates, with faster decay observed at 20 °C. In contrast, enveloped viruses generally exhibit shorter survival times, ranging from hours to days, depending on surface type and environmental conditions. Bean et al. (1982) demonstrated that influenza A and B remain viable for 24–48 h on hard, non-porous surfaces such as stainless steel and plastic, with transfer to hands possible for up to 24 h. However, survival on porous materials was limited to 8–12 h, and hand transferability lasted only 15 min. Greatorex et al. (2011) further showed that while the influenza virus can persist on non-porous surfaces, infectivity typically declined within 4–9 h under indoor conditions and rarely persisted beyond 9 h, highlighting the combined influence of humidity, surface type, and organic material on viral survival.

The adhesion mechanisms of SARS-CoV-2 involve electrostatic, hydrophobics, and van der Waals forces, with protein E enhancing stability in alkaline pH conditions (Vasickova et al., 2010; Siddiquie et al., 2020). While studies documented SARS-CoV-2 environmental contamination, direct evidence of surface transmission is lacking (Brief, 2020). Close contact often does not facilitate distinguishing fomite from respiratory droplet transmission (Brief, 2020). However, consistent findings of surface contamination near infected individuals suggest that fomite transmission remains a plausible pathway for SARS-CoV-2 (Brief, 2020). Boone and Gerba (2005) reviewed the significance of respiratory and enteric virus transmission via fomites (Boone and Gerba, 2005). Compelling studies demonstrated surface contribution to respiratory virus transmission (Boone and Gerba, 2007). For instance, in a study using an aerosolized source, human parainfluenza virus 1 (HPIV1) infected only 2 out of 40 children at a distance of 60 cm, suggesting that aerosol transmission was limited. However, transmission may occur through surface contamination or close contact (Hendrickson, 2003). While direct evidence of fomite transmission is limited, studies like that of Gwaltney Jr and Hendley (1982) provide indirect evidence (Gwaltney Jr and Hendley, 1982). Their research showed that 50% of subjects developed infections after handling a rhinovirus-contaminated coffee cup, and self-inoculation by rubbing the nasal mucosa with contaminated fingers was a likely route of transmission (Gwaltney Jr and Hendley, 1982). Enteric viruses, such as rotavirus, astrovirus, calicivirus, and HAV, primarily spread through the fecal-oral route (Keswick et al., 1983; Ward et al., 1995; Abad et al., 2001; Boone and Gerba, 2007; Weber et al., 2010; Abad et al., 1994). Surfaces can become contaminated through vomit or feces from infected individuals, contributing to the spread of viruses (Keswick et al., 1983; Ward et al., 1995; Abad et al., 2001; Abad et al., 1994; Barker et al., 2004; Weber et al., 2010). Direct evidence of transmission includes a study where 63 to 100% of volunteers became infected after touching rotavirus-contaminated surfaces and then licking their fingers (Keswick et al., 1983; Ward et al., 1995; Abad et al., 2001; Weber et al., 2010; Tuladhar et al., 2013).

##### 3.1.3.3 Fungi

The persistence of fungi such as *Candida* and *Aspergillus* on hospital surfaces ranges from several days to several months, with factors including surface type, humidity, organic load, and biofilm formation playing a critical role in prolonging their survival (Wißmann et al., 2021; Jabłońska-Trypuć et al., 2022; Kramer et al., 2024). Fungal adhesion to surfaces is mediated by proteins such as Als adhesins in *Candida* and hydrophobins in *Aspergillus*, which facilitate persistence on both biotic and abiotic surfaces (Kumari et al., 2021; Subroto et al., 2022). This adhesive capacity, combined with their ability to form biofilms, contributes significantly to their prolonged survival in contaminated environments.

Nosocomial fungi, including *C. albicans*, pose a significant risk in healthcare settings due to their ability to survive for extended periods on dry surfaces. *C. albicans* can persist for up to 4 months on hospital surfaces, contributing to HAIs (Kramer et al., 2024). This fungus exhibits a broad tolerance range, thriving in a wide range of temperatures from 0 °C to 60 °C and pH levels (between 2 and 10) (Sherrington et al., 2017), with their growth being particularly enhanced on wet surfaces (Jabłońska-Trypuć et al., 2022). This factor influences their persistence in healthcare environments, where moisture is often present (Jabłońska-Trypuć et al., 2022). The ability of *Candida* spp. to form biofilms protects them from disinfectants and antifungal treatments, particularly in immunocompromised patients (Wißmann et al., 2021), making them difficult to eradicate from hospital environments, increasing the potential for transmission.

The resilience of *Aspergillus* spp. relies on their ability to produce durable spores primarily disseminated through contaminated air systems (Gerson et al., 1994; Cristina et al., 2009; Park et al., 2019; Jabłońska-Trypuć et al., 2022).

### 3.2 Environmental parameters

Environmental conditions, including temperature, relative humidity (RH), pH, nutrients, and UV radiation (Memarzadeh, 2012; Wißmann et al., 2021; Lopez et al., 2013; Jabłońska-Trypuć et al., 2022; Porter et al., 2024), are critical modulators of pathogen persistence on inanimate surfaces. The complex interactions between these factors significantly influence survival patterns across different microbial species.

Temperature and moisture conditions exhibit complex interdependent relationships with microbial persistence. Lower temperatures (4–8 °C) generally promote extended survival across microbial species, with the protective effect enhanced by specific humidity conditions that mitigate desiccation stress (Williams et al., 2005; Kramer et al., 2006; Kim et al., 2008; Levin et al., 2009; Thompson and Bennett, 2017; Wißmann et al., 2021). High temperatures (>30 °C) typically reduce persistence through multiple mechanisms, including decreased enzymatic activity and structural damage to cellular components. The sensitivity to temperature varies by organism type (Kramer et al., 2006; Choi et al., 2014; Jabłońska-Trypuć et al., 2022; Kramer et al., 2024).

The influence of moisture, encompassing RH and environmental water content, reveals distinct patterns across microbial groups based on their structural characteristics. Humid environments are increasingly hot spots for the persistence of GNB, including MDR pathogens (Kramer et al., 2006; Hassan et al., 2025; Hayward et al., 2025; Hennebique et al., 2025). GPB, with their robust cell wall architecture, demonstrate superior tolerance to moisture stress compared with GNB (Kramer et al., 2024; Jabłońska-Trypuć et al., 2022). Moisture requirements show species-specific optimization. Studies showed that *Pseudomonas* spp., *Klebsiella* spp., and *Enterobacter* spp. thrive under higher RH (>70%) combined with lower temperatures, while *E. coli*, *Proteus vulgaris*, and certain *Salmonella* spp. show reduced viability at intermediate RH (50–70%) (Jabłońska-Trypuć et al., 2022). Viral persistence patterns correlate strongly with virion structure, with enveloped viruses demonstrating optimal preservation of infectivity at low RH (20–30%), particularly at reduced temperatures (Aboubakr et al., 2021; Zeng et al., 2023; Kramer et al., 2024), while non-enveloped viruses show enhanced stability at higher RH (70–90%; Kramer et al., 2024; Jabłońska-Trypuć et al., 2022). However, individual studies reveal contradictions to these general patterns. Non-enveloped RNA viruses, such as rotavirus, persist longer under low relative humidity (25–50%) and cooler temperatures (4 °C), with approximately 10% infectivity remaining after 10 days, whereas survival declines to <1% within 48 h at 22 °C and high RH (85%; Sattar et al., 1986). Moe and Shirley (1982) reported that rotavirus survival was longer at both low and high RH compared with intermediate levels, but also that infectivity decreased more rapidly at elevated temperature (37 °C) than at 4 or 20 °C, indicating that temperature can override humidity effects. Fungal species generally exhibit optimal persistence in moisture-rich environments, with enhanced biofilm formation capabilities under these conditions (Jabłońska-Trypuć et al., 2022). Moisture-dependent persistence patterns may not fully reflect the behavior of microorganisms embedded within biofilms, as these structures generate a protected microenvironment that buffers microbial survival from ambient conditions (Donlan, 2000; Donlan, 2002; Kramer et al., 2024; Schapira et al., 2024). The extracellular polymeric matrix retains water and nutrients, shielding embedded organisms from desiccation and low RH that would otherwise reduce viability (Donlan, 2000; Donlan, 2002; Kramer et al., 2024; Schapira et al., 2024). Beyond passive protection, biofilms function as highly organized, self-sufficient communities that facilitate metabolic cooperation, enhance resistance to stressors, and adhere robustly to both abiotic and biotic surfaces. These interactions collectively extend microbial persistence and complicate eradication in healthcare environments (Flemming et al., 2016).

This phenomenon is particularly relevant for dry surface biofilms documented on hospital equipment (Costerton et al., 1995b; Kramer et al., 2024). Pathogens such as *K. pneumoniae* remain viable for weeks at 55% ± 5% relative humidity at room temperature (21 °C) (Costerton et al., 1995b; Kramer et al., 2024). By prolonging resistance to desiccation and conferring up to 500-fold increased tolerance to disinfection (Costerton et al., 1995b; Costerton, 1995a; Centeleghe et al., 2023; Kramer et al., 2024), biofilms may extend the window for fomite-borne transmission beyond what planktonic persistence studies predict. Importantly, the quantitative contribution of biofilms to cross-transmission events in healthcare settings remains unresolved, representing a critical gap for evidence-based surface decontamination protocols (Kramer et al., 2024).

Biological and organic sources confer environmental moisture, adding another layer of complexity to persistence patterns. The presence of secretions, excretions, and organic materials provides protective effects and nutritional support that can significantly extend survival times (Abad et al., 1994; Rabenau et al., 2005; Memarzadeh, 2012; Aboubakr et al., 2021; Kramer et al., 2024). These conditions facilitate the formation of complex ‘collective infectious units’ that enhance transmission potential (Kramer et al., 2024). For example, cytomegalovirus (CMV) demonstrates prolonged infectivity on absorbent surfaces in the presence of saliva, highlighting the role of biological fluids in modulating persistence (Stowell et al., 2012). This protective effect of organic materials extends across viral, bacterial, and fungal species, often leading to enhanced survival even under otherwise suboptimal temperature and humidity conditions.

Light, particularly sunlight or UV radiation, significantly influences the survival and stability of pathogens on surfaces, representing a critical factor in environmental behavior (especially outdoor) but also in decontamination and infection control (Wißmann et al., 2021; Jabłońska-Trypuć et al., 2022; Kramer et al., 2024). Various studies have demonstrated how light conditions can alter pathogen viability, with many microorganisms showing increased susceptibility to inactivation under light exposure through both direct photochemical damage and indirect oxidative stress mechanisms (Wißmann et al., 2021; Jabłońska-Trypuć et al., 2022; Kramer et al., 2024).

For instance, Thompson and Bennett (2017) conducted a comprehensive investigation into the survival of Influenza A virus strains on different surfaces (cotton, microfiber, and stainless steel) under controlled conditions, including light or not (Thompson and Bennett, 2017). The study revealed that all strains survived for up to two weeks on stainless steel in a dark environment, whereas survival on microfiber and cotton demonstrated higher variability due to surface porosity and moisture retention characteristics (Memarzadeh, 2012; Thompson and Bennett, 2017; Wißmann et al., 2021). The survival times in light conditions were comparable to those in the dark, suggesting that factors beyond light exposure, such as surface material properties, environmental humidity, and temperature fluctuations, play the most crucial roles in viral persistence (Thompson and Bennett, 2017). It should be noted, however, that the authors did not specify whether the “light” condition in their study referred to natural sunlight or artificial laboratory lighting, nor did they report light intensity, spectrum, or photoperiod (Thompson and Bennett, 2017).

Natural sunlight has a detrimental effect on the survival of viruses, bacteria, and fungi on surfaces, primarily through its UV components and associated photochemical reactions (Stowell et al., 2012; Ratnesar-Shumate et al., 2020; Jabłońska-Trypuć et al., 2022). The microbial species and the surface characteristics significantly influence the degree to which light affects survival, with variations observed across different taxonomic groups and surface materials.

Notably, *Mycobacterium tuberculosis* exhibits a marked difference in survival depending on natural light conditions, with the pathogen surviving for merely 2–5 days in daylight but remaining viable for up to 200 days in dark conditions (Kramer et al., 2024). This dramatic contrast highlights the potential importance of natural light exposure in infection control strategies, particularly in healthcare settings. The survival of fungal species, such as *C. albicans* and *Rhodotorula rubra*, on glass surfaces extended when kept in dark conditions, with survival times nearly doubling compared with those kept in daylight, confirming the critical role of light exposure in microbial persistence and viability (Kramer et al., 2024).

In general, pathogens exposed to light, particularly ultraviolet radiation, experience accelerated degradation and inactivation through multiple mechanisms, including direct DNA/RNA damage and the generation of reactive oxygen species, thereby reducing their transmission potential (Agodi et al., 2007; Nseir et al., 2011; Mitchell et al., 2015). Conversely, the absence of light allows for extended survival periods, enhancing their ability to persist and spread in environmental reservoirs (Agodi et al., 2007; Nseir et al., 2011; Mitchell et al., 2015). These findings have significant implications for infection control protocols, particularly in healthcare settings where managing pathogen survival on surfaces is crucial for preventing HAIs.

The pH of the environment plays a fundamental role in determining microbial survival and persistence on surfaces (Kramer et al., 2024; Jabłońska-Trypuć et al., 2022). Different microorganisms have evolved distinct mechanisms to cope with varying pH conditions, leading to significant differences in their survival capabilities.

For viruses, pH-dependent behavior has been documented for specific viruses rather than across all viral families. SARS-CoV-2, for instance, demonstrates stability across a broad pH spectrum with notably enhanced stability under alkaline conditions (Chin et al., 2020). This pH preference is partly mediated by protein E, a component of the virus’s hydrophobic layer that facilitates adhesion to surfaces (Raiteux et al., 2021; Rathnasinghe et al., 2021). The relationship between pH and viral load on surfaces is well-documented, with environmental pH directly influencing the concentration of viable viral particles (Murphy and Brauer, 2011; Chin et al., 2020).

Among bacteria, *Haemophilus influenzae* employs urease expression as a survival mechanism in acidic environments during respiratory tract infection, illustrating species-specific adaptations to pH stress (Murphy and Brauer, 2011; Jabłońska-Trypuć et al., 2022). This enzyme allows the bacterium to modify its local environment when exposed to low pH conditions (Murphy and Brauer, 2011), effectively creating a more favorable microenvironment for survival. This adaptation is particularly relevant in clinical settings with varying pH conditions.

Nosocomial fungi exhibit some of the broadest pH tolerances among healthcare-associated pathogens. These microorganisms can thrive in environments with a pH ranging from 2.0 to 8.5 (Chandra et al., 2001; Davis, 2003; Vasickova et al., 2010; Kobayashi and DeLeo, 2013). This extensive pH tolerance and ability to adapt to various moisture conditions contribute to their persistence as significant healthcare-associated pathogens (Kramer et al., 2006; Weber et al., 2013a,b; Otter et al., 2013).

### 3.3 Surface characteristics and cleanability

Surface characteristics influence bacterial persistence through complex interactions between microbial physiology and environmental conditions (Robine et al., 2000; Jabłońska-Trypuć et al., 2022; Kramer et al., 2024; Porter et al., 2024). The interface where a solid surface contacts a liquid environment (such as water or blood) provides favorable conditions for microorganisms to adhere and proliferate (Donlan, 2002). Multiple factors can influence microbial attachment, including the nature of the underlying surface, the conditioning biofilms on that surface, the flow patterns of the liquid, the properties of the liquid itself, and the distinctive features of the microbial cell surfaces (Donlan, 2002). Porous and non-porous surfaces create distinct microenvironments that modulate microbial survival through varying mechanisms of moisture retention, surface energy, and biofilm development potential (Firquet et al., 2015; Berne et al., 2015; Berne, 2023; Jin and Sengupta, 2024). Beyond porosity, microbial adhesion is further affected by additional surface properties, including charge distribution, wettability, topography, roughness, and mechanical stiffness (Abad et al., 1994; Jabłońska-Trypuć et al., 2022; Zheng et al., 2021; Sherry et al., 2022; Reyes et al., 2022; Porter et al., 2024).

Non-porous surfaces such as stainless steel, glass, and plastic provide stable environments that support extended microbial viability by maintaining consistent hydration and facilitating robust biofilm development (Porter et al., 2024). A study measured the survival of *Pseudomonas fluorescens* and *Enterococcus faecalis* on glass, polyvinyl chloride (PVC), and stainless-steel surfaces (Robine et al., 2000). Bacterial suspensions were prepared in filtered deionized water before aerosolization and deposition onto the test surfaces (Robine et al., 2000). The highest lethality was measured on PVC, followed by glass and steel (Robine et al., 2000). This effect was linked to the polymer’s high reactivity with the presence of oxidizing sites and additives, and the release of hydrochloric acid (HCl), leading to 90% inactivation of *E. faecalis* within 96 h at 0% RH (Robine et al., 2000). The survival differences between materials were explained by surface properties: glass possesses an adsorbed water layer that exerts a depressant (negative) effect on bacterial cells, whereas stainless steel appears relatively inert, promoting longer bacterial survival (Robine et al., 2000). Conversely, porous surfaces create microenvironments with variable nutrient availability and protective characteristics, allowing bacterial cells to exploit moisture gradients and find protective niches within fiber layers. The survival of specific bacterial strains, including *S. aureus*, *P. aeruginosa*, and *E. coli*, demonstrates adaptability, with persistence times ranging from hours to months depending on surface composition, moisture content, and environmental parameters (Jabłońska-Trypuć et al., 2022). Bacterial survival represents a sophisticated interplay of surface characteristics, microbial adaptation, and environmental conditions (Robine et al., 2000; Kramer et al., 2006).

Viruses demonstrate remarkably complex survival strategies that vary significantly based on surface characteristics and viral structural properties. Viruses can be detected for a longer period on porous compared with non-porous surfaces (Kramer et al., 2024). The prolonged survival of viruses can be attributed to several mechanistic factors (Kramer et al., 2024; Porter et al., 2024). One significant explanation is the reduced efficiency of virus elution during recovery from porous substrates (Kramer et al., 2024; Porter et al., 2024), meaning that viral particles become more deeply embedded within the microstructure of these materials, making them less prone to dislodgment and subsequent detection. Furthermore, the capillary effect inherent to the microscopic cavities of porous surfaces may create a microenvironment that offers physical and chemical protection to the viruses, shielding them from external stressors such as ultraviolet radiation and desiccation (Kramer et al., 2024). The relationship between material porosity and viral persistence is complex, with seemingly contradictory findings in the literature. As evidenced by Huang et al. (2025), the accelerated evaporation of aerosols on porous surfaces may stabilize certain viruses, particularly non-enveloped viruses inherently resistant to drying, by establishing conditions that slow their inactivation (Huang et al., 2025). Huang’s findings further suggest that porous materials can also trap viruses within their structure, potentially protecting them from environmental stressors. Non-enveloped viruses often persist longer on porous materials compared to non-porous surfaces, whereas many enveloped viruses show reduced persistence on porous materials, though exceptions exist. These observations highlight that material porosity alone does not determine viral persistence, but rather interacts with virus structure, specific strain characteristics, and environmental conditions (Huang et al., 2025).

When a respiratory droplet dries on a surface, a residual thin-liquid film remains and evaporates slowly due to disjoining pressure (Chatterjee et al., 2021; Chatterjee et al., 2022). This slow evaporation allows viruses to survive for extended periods. Virus aggregation may add complexity to the survival mechanism depending on the viral type, solution salts, and pH (Gerba and Betancourt, 2017). Lipid-enveloped viruses exhibit pronounced survival differences between porous and non-porous surfaces (Robine et al., 2000; Kramer et al., 2024; Porter et al., 2024). Studies showed that at RH ranging between 35 and 40%, *Influenza A* virus can remain viable for up to 48 h on non-porous surfaces like stainless steel and plastic, but only a few hours on porous materials like cloth or paper (Bean et al., 1982). This differential survival is due to the surface’s capacity to maintain viral particles in a stable, hydrated microenvironment for preserving viral infectivity. For example, non-porous surfaces provide a uniform, moisture-retentive environment that protects the structural integrity of viruses (Chatterjee et al., 2021). The viral envelope’s lipid membrane remains stable when not subjected to rapid moisture absorption, which can disrupt protein conformations and compromise the virus functionality (Firquet et al., 2015; Zeng et al., 2023). SARS-CoV-2 exemplifies this phenomenon, maintaining viability for up to 72 h on non-porous surfaces while exhibiting a significant reduction on porous materials, where absorption and desiccation rapidly inactivate viral particles (Chin et al., 2020).

The described mechanisms are not mutually exclusive and coexist. Their relative importance depends on factors such as the type of virus, the specific properties of the material, and environmental conditions like humidity and temperature. For example, in environments with high humidity, the protective microenvironment might play a more significant role, potentially extending viral survival. In contrast, in dry conditions, accelerated thin-film evaporation might dominate, reducing viral survival time (Firquet et al., 2015; Owen et al., 2021; Chatterjee et al., 2021; Chatterjee et al., 2022; Zeng et al., 2023). Therefore, the interplay between viral structure, surface characteristics, and environmental conditions reveals viral survival as a sophisticated, context-dependent process. Surface porosity, moisture content, temperature, and humidity collectively modulate viral persistence, challenging simplistic models of viral transmission.

Non-enveloped viruses, including norovirus and rotavirus, demonstrate exceptional resilience across surface types. These viruses possess robust protein capsids that resist environmental degradation and persist for days to weeks on various surfaces such as countertops, doorknobs, and tiles [2].

Understanding the relationship between environmental factors and media composition is critical for examining the persistence and risks of viral transmission in real-life settings (Firquet et al., 2015). Laboratory studies often use standardized cell culture media, sometimes supplemented with fetal calf serum (FCS) to mimic biological conditions, which may not accurately represent real-world scenarios. Viruses in clinical situations are found in diverse media, ranging from protein-rich fluids such as serum to protein-poor environments, such as water (Zheng et al., 2021).

Fungal pathogens exhibit varied persistence on porous and non-porous surfaces, significantly influencing their potential for nosocomial transmission. On non-porous surfaces, *C. auris* showed higher survival (~93%) on moist agar under nutrient-deprived conditions and maintained viability on dry stainless steel for up to 7 days, with approximately 38% survival (Piedrahita et al., 2017; Kramer et al., 2024). A study showed that *Candida parapsilosis* survives better than *C. albicans* on non-porous surfaces. Porous substrates, such as textiles, provide microenvironments within their cavities, shielding pathogens from environmental stressors like desiccation and UV radiation. *C. albicans* survived on textiles significantly better than on glass and metal, while *C. parapsilosis* survives on fabrics and non-porous supports (Traoré et al., 2002; Jabłońska-Trypuć et al., 2022; Dixit et al., 2023). However, direct comparisons between studies are complicated by variations in experimental conditions, including differences in inoculum preparation, temperature, relative humidity, exposure duration, and recovery methods. These methodological disparities can significantly influence reported persistence outcomes and should be considered when interpreting findings across different fungal species and surface types.

## 4 Inanimate surface-born microbial transmission and infectious risk

### 4.1 Research approaches

Substantial evidence showed that inanimate surface transmission to humans is a critical pathway (Stephens et al., 2019; Jin and Sengupta, 2024). Surface-mediated microbial transmission has been extensively studied through multiple methodological approaches, including epidemiological investigations, experimental measurements, and mathematical modeling.

Environmental surveillance studies have demonstrated the widespread presence of pathogens. across diverse settings, highlighting the role of fomites in disease transmission. Norovirus and influenza A virus have been detected on high-touch surfaces in schools, while respiratory viral. RNA, particularly picornavirus, was found on 20% of pediatric waiting room toys (Pappas et al., 2010; Bright et al., 2010). In healthcare facilities, human adenoviruses were identified in 45% of ICU fomite samples, with viral loads ranging from 2.48 × 10^1^ to 2.1 × 10^3^ genomic copies per milliliter, and SARS-CoV-2 RNA was detected on 30% of hospital surfaces (Dowell et al., 2004; Ganime et al., 2014). Similarly, MERS-CoV was identified on 4% of high-touch surfaces in patient rooms, and environmental contamination was documented in 54% of households with individuals colonized with the virus (Knox et al., 2012; Khan et al., 2016). Additionally, studies have shown that mobile phones and computer keyboards in ICUs are unlikely reservoirs for multidrug-resistant organisms, while healthcare attire and devices have been implicated as potential fomites, emphasizing the need for stringent hygiene practices (Haun et al., 2016; Smibert et al., 2018). These findings collectively underscore the crucial role of environmental surveillance and infection control measures in mitigating the spread of infectious agents. Experimental studies have provided robust evidence on microbial transfer mechanisms from fomites, highlighting the role of surface contamination in pathogen transmission. Human norovirus transfer efficiency from contaminated finger pads to surfaces and food items initially ranged from 13%, declining over time and with surface drying, while infectious virus transfer to finger pads averaged 2–4% after 40 min (Weber et al., 2010; Tuladhar et al., 2013). MRSA transfer rates varied between 0 and 20% across glove materials and fomites, influenced by glove type and body fluid adsorption (Moore et al., 2013), while *A. baumannii* transfer efficiencies were reduced by 50% with latex gloves (Greene et al., 2015). Rhinovirus contamination was detected on 35% of hotel room surfaces and 60% of fingertip samples after intentional transfer activities (Winther et al., 2007). Influenza viruses survived up to 48 h on hard surfaces, with transfer to hands possible for 24 h post-deposition (Bean et al., 1982). Transfer efficiencies were higher under high relative humidity, particularly for non-porous surfaces [14], with GNB exhibiting transfer rates of 41% (Rusin et al., 2002). Phage recovery from door handles ranged from 30 to 66% (Rheinbaben et al., 2000), and DNA surrogate viruses demonstrated 100% transfer rates in controlled settings (Jiang et al., 1998). Epidemiological studies have provided crucial insights into fomite-mediated transmission, though they face inherent methodological challenges. Observational cohort studies often struggle to distinguish the specific contribution of fomites from other exposure routes such as direct contact or aerosols (Weber et al., 2013a,b; Stephens et al., 2019). Natural experiments, particularly disease outbreaks, offer better opportunities to identify transmission pathways (Stephens et al., 2019). Stephens et al. (2019) reported two studies to substantiate these findings (Stephens et al., 2019). In a recurring norovirus outbreak on an airplane, flight crews across several subsequent shifts became ill up to 5 days after an infected passenger vomited. The problem ceased once new carpets were placed in the plane (Thornley et al., 2011), providing strong evidence for fomite transmission of norovirus (Thornley et al., 2011; Weber et al., 2013a,b; Stephens et al., 2019). In contrast, fomite-mediated transmission of the influenza virus is less well understood. For instance, during an outbreak on another airplane, 54 passengers were exposed to an infected individual during a 3-h ground delay with no ventilation, resulting in 72% of passengers developing symptoms (Moser et al., 1979; Stephens et al., 2019), demonstrating the difficulty of clearly concluding on fomite transmission.

A norovirus outbreak on Arizona houseboats showed heavy contamination of indoor surfaces, especially in bathrooms and kitchens (Jones et al., 2007). This finding suggests that surface contamination is the main route of norovirus spread in this confined environment, highlighting the need for effective cleaning to prevent future outbreaks. Despite such data, Kutter et al. (2018) noted that many studies still do not conclusively prove transmission routes.

Evidence from epidemiology studies establishes links between many viruses and fomite transmission. Virus outbreaks, including smallpox, influenza, SARS, rotavirus, hepatitis A, RSV, and norovirus, have all been tied to contaminated surfaces in locations such as nursing homes, daycares, hospitals, and public places. Healthcare-associated infections (HAIs) are a source of patient morbidity and mortality, with approximately 20 to 40% linked to cross-infection via healthcare workers’ hands, often after contact with surfaces contaminated with *norovirus, C. difficile*, and *Acinetobacter* spp. (Weber et al., 2010).

These findings indicate that epidemiological evidence supports surfaces as important vectors in both community and healthcare settings.

Mathematical modeling studies have provided theoretical frameworks for understanding the modalities of fomite-mediated transmission. Mathematical and mechanistic models further advance our understanding by providing “proof of principle” for the involvement of contaminated surfaces in disease transmission (Otter et al., 2013; Stephens et al., 2019). Quantitative microbial risk assessments (QMRA) utilize data on microorganism abundance on fomites, human-fomite interaction rates, and microbial transfer efficiencies to quantify potential health risks (Stephens et al., 2019). These models often incorporate detailed mechanistic elements, such as Markov chain models combined with mass balance or computational fluid dynamics (CFD) models, to simulate physical transport and pathogen dynamics within built environments (Stephens et al., 2019).

Kraay et al. (2018) developed compartmental models between direct-fomite and hand-fomite transmission, demonstrating significant transmission potential for rhinovirus and norovirus while suggesting lower sustainability for influenza virus through fomites alone (Kraay et al., 2018). Cowling et al. (2013) used mathematical modeling to analyze influenza A transmission and estimated that long-range aerosol transmission contributed to about half of all transmission events.

More recently, modeling has been applied in healthcare epidemiology and infection prevention, where surveillance data and mechanistic models are used to forecast healthcare impact, evaluate IPC interventions, and guide antimicrobial stewardship decisions (Grant et al., 2024). System-level models have further shown that healthcare collapse, with limited resources and weakened infection control, significantly increases the risk of nosocomial infection by raising the reproduction number of hospital-acquired pathogens (Shaale et al., 2025). Advances in computational forecasting, such as frequency-aligned spectral filtering, have improved long-range influenza predictions, supporting hospital preparedness, vaccination strategies, and resource allocation (Feng et al., 2025). Beyond academic studies, commercial platforms now embed modeling into operational infection control systems. Commercial platforms are increasingly embedding mathematical modeling into infection control workflows, enabling real-time monitoring of disinfection practices and supporting outbreak prevention strategies. Looking ahead, Artificial Intelligence (AI) and Internet of Things (IoT) technologies are expected to further enhance infection control workflows, enabling real-time monitoring of disinfection practices, predictive risk assessment, and automated outbreak alerts.

## 5 Discussion

The comprehensive investigation of microbial contamination across diverse environmental interfaces represents a critical scientific frontier with profound implications for infection prevention and control. Surfaces play a key role in the transmission chain as reservoirs, enabling bidirectional pathogen transfer between hands (skin), secretions and surfaces, amplifying infection risks (Salmanov et al., 2023; Elkady et al., 2022; Hu et al., 2015). This evidence shows that surface contamination should not be seen as isolated events. These are systemic processes that require integrated prevention strategies.

Among the different ways to impact the transmission chain, effective cleaning and disinfection protocols, particularly those incorporating training, regular auditing, and feedback mechanisms, have proven most effective when implemented as part of a “bundle” approach (Browne and Mitchell, 2023). Multimodal cleaning strategies that combine environmental cleaning/disinfection with collaboration among care staff have been identified as the most effective interventions (Société Française d'Hygiène Hospitalière, 2017; World Health Organization, 2018; Centers for Disease Control and Prevention, 2019; Ministère de la Santé Francaise, 2019; Institut Pasteur de Lille, 2023; Haute Autorité de Santé, 2025). Effective cleaning is essential for the removal of bacterial spores, including *C. difficile*, which are resistant to many disinfectants. However, for vegetative cells, which are more susceptible to biocidal agents, disinfection plays the primary role in reducing contamination and preventing infection.

Advances in disinfectant technologies and antimicrobial surfaces highlight opportunities but also limitations. While continuous disinfection and non-leaching properties promise prolonged efficacy and safety (Jabłońska-Trypuć et al., 2022; Feuillolay et al., 2018; Iskandar et al., 2023; Roquefeuil et al., 2024; Iskandar et al., 2025), real-world validation remains limited. IPC strategies should therefore prioritize technologies that are sustainable, non-toxic, and adaptable to diverse healthcare settings, particularly in resource-constrained environments. Spatial design strategies such as pressure-controlled isolation rooms, anterooms for protective equipment, and clean/dirty pathways provide reliable preventive measures that disrupt microbial transmission (Wohrley and Bartlett, 2019; Gibbons, 2016). Embedding IPC principles into facility design ensures resilience against outbreaks, complementing behavioral and cleaning interventions. In parallel, antimicrobial surfaces represent a material-level innovation that can reduce contamination risks when evaluated for real-world efficacy, durability, and safety (Cunliffe et al., 2021; Colin et al., 2020; Gómez-Mejia et al., 2025; Tarabal et al., 2025). Together, infrastructure design and surface technologies highlight that IPC must extend beyond cleaning practices to encompass the built environment and material science, ensuring sustainable and safe protection against microbial transmission.

Global regulatory frameworks, such as the EU Biocidal Products Regulation (Regulation (EU) No 528/2012) (European Commission, n.d.) and comparable systems in the US (FIFRA), UK (UK BPR), Japan (CSCL, METI), and Australia (APVMA), emphasize rigorous evaluation of safety and efficacy, often banning chemicals when safer alternatives exist. These policies highlight the importance of balancing antimicrobial efficacy with human and environmental safety, while ensuring innovation aligns with public health priorities (Jabłońska-Trypuć et al., 2022; Iskandar et al., 2023).

At the systemic level, limited access to timely care, sanitation, and protective resources heightens vulnerability to surface-mediated infections, particularly among marginalized populations (Tarabay et al., 2024; Sachs et al., 2022; Rad, 2025). While the WHO’s universal health coverage agenda highlights equity as indispensable (Ahmed et al., 2025a; Ahmed et al., 2025b; World Health Organization, 2025), disparities persist between high-income countries with robust IPC systems and low- and middle-income countries (LMICs) with fragile infrastructures (Tosam et al., 2019; Tarabay et al., 2024; Tartari et al., 2026; Tartari et al., 2021). These inequities, compounded by colonial legacies (Rad, 2025) and emerging challenges such as climate change and the digital divide (Abi Deivanayagam et al., 2023), indicate that microbial contamination is not only a biomedical issue but also a social and structural one.

This perspective aligns directly with SDG 3 (Good Health and Well-Being), particularly its universal health coverage target, which emphasizes equity and “health for all.” It also intersects with SDG 10 (Reduced Inequalities), as equitable access to healthcare and sanitation is essential for mitigating surface-mediated transmission risks. Addressing microbial contamination thus contributes to global health security while advancing broader equity and sustainability agendas (United Nations, 2015).

Future research should prioritize:

- Refining methods for detecting viable but non-culturable organisms and biofilm persistence (Bjarnsholt et al., 2018; Bridier et al., 2012; Kramer and Assadian, 2014; Almatroudi et al., 2015; Vestby et al., 2020; Centeleghe et al., 2023).
- Optimizing cleaning protocols to address persisters and resistant spores (Jabłońska-Trypuć et al., 2022).
- Validating novel technologies such as automated disinfection and antimicrobial surfaces under real-world conditions (Feuillolay et al., 2018; Iskandar et al., 2023; Roquefeuil et al., 2024; Gómez-Mejia et al., 2025; Tarabal et al., 2025).
- Developing predictive models that integrate molecular sequencing, computational approaches, and longitudinal studies to anticipate transmission risks across healthcare, domestic, and public settings (Mitchell et al., 2015; Wißmann et al., 2021; Kramer et al., 2024; Porter et al., 2024; Dunn et al., 2013).
- These efforts will strengthen IPC and ensure that innovation aligns with equity, sustainability, and global health priorities.

## 6 Conclusion

The ongoing study of fomite-borne pathogen transmission highlights its importance and the urgent need for experimental designs that more closely reflect the realities of healthcare environments. While significant strides have been made in understanding pathogen behavior under controlled conditions, the complexities of real-world contamination, including multiple pathogen interactions (with other microorganisms and biotic/abiotic surfaces) and environmental variables, remain always poorly understood. Future research should prioritize the development of sophisticated experimental models that simulate endemic environments, account for moisture-related factors such as humidity, and include diverse contamination states. The integration of real-time tracking technologies, advanced molecular tools, and interdisciplinary approaches is crucial in capturing the full dynamics of microbial ecosystems and transmission. Longitudinal and multi-site studies focusing on pathogen persistence, the detection of VBNC cells and their role in colonization/infection risks, and the development of predictive transmission models will be essential for advancing infection control strategies. The objective is improving known preventive measures already identified as capable of interrupting or limiting the chain of transmission as cleaning and disinfection protocols. Currently, the effectiveness of these measures is widely recognized, but the risk remains, and reducing it requires research and development of new concepts for the limitation of surface contamination and transmission risk, and enhancing our overall understanding of microbial resilience in healthcare and public environments.
